# Reliability of the freehand region-of-interest method in quantitative cerebral diffusion tensor imaging

**DOI:** 10.1186/s12880-021-00663-8

**Published:** 2021-10-04

**Authors:** Ullamari Hakulinen, Antti Brander, Tero Ilvesmäki, Mika Helminen, Juha Öhman, Teemu M. Luoto, Hannu Eskola

**Affiliations:** 1 Department of Medical Physics, Medical Imaging Center of Pirkanmaa Hospital District, Tampere, Finland; 2 Department of Radiology, Medical Imaging Center of Pirkanmaa Hospital District, Tampere, Finland; 3 Faculty of Medicine and Health Technology, Tampere University, Tampere, Finland; 4 Faculty of Social Sciences, Health Sciences, Tampere University, Tampere, Finland; 5 Tays Research Services, Tampere University Hospital, Tampere, Finland; 6 Department of Neurosurgery, Tampere University Hospital and Tampere University, Tampere, Finland

**Keywords:** Diffusion tensor imaging, Reliability, Repeatability, Intra-class correlation coefficient, Fractional anisotropy, Apparent diffusion coefficient, Axial diffusivity, Radial diffusivity, ROI-based method, Freehand method

## Abstract

**Background:**

Diffusion tensor imaging (DTI) is a magnetic resonance imaging (MRI) technique used for evaluating changes in the white matter in brain parenchyma. The reliability of quantitative DTI analysis is influenced by several factors, such as the imaging protocol, pre-processing and post-processing methods, and selected diffusion parameters. The region-of-interest (ROI) method is most widely used of the post-processing methods because it is found in commercial software. The focus of our research was to study the reliability of the freehand ROI method using various intra- and inter-observer analyses.

**Methods:**

This study included 40 neurologically healthy participants who underwent diffusion MRI of the brain with a 3 T scanner. The measurements were performed at nine different anatomical locations using a freehand ROI method. The data extracted from the ROIs included the regional mean values, intra- and inter-observer variability and reliability. The used DTI parameters were fractional anisotropy (FA), the apparent diffusion coefficient (ADC), and axial (AD) and radial (RD) diffusivity.

**Results:**

The average intra-class correlation coefficient (ICC) of the intra-observer was found to be 0.9 (excellent). The single ICC results were excellent (> 0.8) or adequate (> 0.69) in eight out of the nine regions in terms of FA and ADC. The most reliable results were found in the frontobasal regions. Significant differences between age groups were also found in the frontobasal regions. Specifically, the FA and AD values were significantly higher and the RD values lower in the youngest age group (18–30 years) compared to the other age groups.

**Conclusions:**

The quantitative freehand ROI method can be considered highly reliable for the average ICC and mostly adequate for the single ICC. The freehand method is suitable for research work with a well-experienced observer. Measurements should be performed at least twice in the same region to ensure that the results are sufficiently reliable. In our study, reliability was slightly undermined by artifacts in some regions such as the cerebral peduncle and centrum semiovale. From a clinical point of view, the results are most reliable in adults under the age of 30, when age-related changes in brain white matter have not yet occurred.

## Background

Diffusion tensor imaging (DTI) is a magnetic resonance imaging (MRI) technique that has become a popular tool for central nervous system imaging [1, 2]. DTI is based on the diffusion characteristics of water molecules, which, in turn, reflect the histological structure of the tissue [3]. Diffusion data can be used to calculate several quantitative parameters, such as fractional anisotropy (FA), the apparent diffusion coefficient (ADC), and axial (AD) and radial (RD) diffusivity. FA indicates the degree of diffusion anisotropy. The diffusion is generally strongest in the orientation parallel to the nerve tracts. The ADC expresses the mean diffusion in each direction. AD can be considered to be modulated by the axonal integrity [4, 5], and its changes can thus reflect the degree of axonal degeneration [6]. RD, on the other hand, is modulated by axonal myelination [4, 5].

Several studies on different neurological diseases have utilized these DTI indices as biomarkers of white matter integrity [7–11]. Significant age-related changes in the integrity of white matter have also been found in healthy volunteers [12–17].

Chronic white matter diseases as well as normal aging, causes a decrease in FA values while RD values tend to increase [18–25]. A strong relationship has also been found between the changes in AD and axonal injury [4]. Moreover, ADC values may temporarily decrease in the acute phase of cerebrovascular accidents, but, in the chronic phase, they usually increase [26, 27].

The imaging process includes several steps between acquisition and the final parametric result, and each step is susceptible to different pitfall sources [28, 29]. Specifically, low resolution, a low signal-to-noise ratio (SNR), and a variety of different types of artifacts can reduce the image quality [30–33]. In particular, the single-shot echo-planar technique used in diffusion imaging can cause severe image distortions because of the long echo trains that are used in the sequence. The consequence of these susceptibility artifacts are geometric distortions at the interfaces between soft tissue and air at the base of the skull [34]. In addition, B_0_ inhomogeneities cause a decrease in the efficiency of fat-saturation pulses [34]. Protons in water and fat have a different Larmor frequency, which leads to fat misregistration in single-shot echo-planar imaging. All of the above-mentioned pitfalls and artifacts also have a detrimental effect on the reliability of parametric results.

Post-processing and analysis methods can be selected according to whether individual or group results are required. The histogram [35], region-of-interest (ROI), and quantitative tractography methods [36] are suitable for both individual- and group-level analysis. In addition, the tract-based spatial statistics (TBSS) method [37] is an option for group analysis. Nowadays, different methods are often used concomitantly, giving additional value to the accuracy of the results [38, 39].

The ROI method is still a highly valid method when measuring individual subjects. While laborious, time-consuming, and observer-dependent, it however, is the most readily available method in commercial clinically approved software. The method can be used to evaluate the focal areas of brain parenchyma of a single subject and it enables leaving artifacts outside the area of measurement. The low or moderate repeatability of the method as well as its high intra- and inter-observer variation have been considered its cons [40].

The main objective of this study was to investigate the reliability of the freehand ROI method, by intra- and inter-observer variation and repeatability measurements. The aim was also to examine the effects of different parameters (FA, ADC, AD and RD) and artifacts on the reliability of the results. In addition, the effects of age on white matter changes were studied in group comparisons.

## Methods

### Subjects

Participants included 40 healthy adult volunteers consisting of 20 women and 20 men with an age range of 18–60 years and a mean age of 40.6 (SD 12.2) years [41, 42]. The age groups were: (i) 18–30, (ii) 31–40, (iii) 41–50, and (iv) 51–60 years. Each age group included five men and five women. Thirty-nine of the subjects were right-handed, and one was left-handed. MRI scans were performed within a year (2010–2011). The exclusion criteria consisted of the following: (i) neurological problems (including abnormalities upon neuroimaging), (ii) psychiatric problems, (iii) history of traumatic brain injury, (iv) former neurosurgical procedure, (v) problems with hearing or vision, (vi) first language other than Finnish, (vii) MRI contraindications, and (viii) refusal to participate. No indications of significant structural abnormalities were found in any of the subjects in conventional clinical sequences. An ethics approval was obtained from the Ethical Committee of the Pirkanmaa Hospital District, and a written consent was obtained from each volunteer.

### MRI acquisition

The subjects were scanned with a 3 T Siemens Trio (Siemens Healthcare, Erlangen, Germany) MRI scanner. The MRI protocol included sagittal T1-weighted 3D IR-prepared gradient echo, axial T2-weighted turbo spin echo, conventional axial and high-resolution sagittal fluid attenuation inversion recovery (FLAIR), axial T2*-weighted, and an axial susceptibility weighted imaging (SWI) series. The DTI data was collected by a single-shot, spin echo-based, and diffusion-weighted echo planar imaging sequence. The parameters for the DTI sequence were the repetition time (TR) 5144 ms, echo time (TE) 92 ms, field-of view (FOV) 230 mm, matrix 128 × 128, 3 averages, slice/gap 3.0/0.9 mm, voxel dimension 1.8 × 1.8 × 3.0 mm^3^, b-factor 0, 1000 s/mm^2^, and 20 diffusion gradient orientations. A 12-channel head coil and a four-channel neck coil were simultaneously used. The coils used in the study were subjected to regular quality tests throughout the study, so that they could be proven to be intact and of high quality.

### Data analysis

The multidirectional diffusion data was visually analyzed for distortions and artifacts. The eddy current distortion was qualitatively estimated by drawing the brain contours to the b_0_ image and copying the contours to the diffusion weighted images. Susceptibility and phase artifacts were verified by reviewing the FA, ADC, AD, RD, and b_0_ maps slice-by-slice.

The SNR was determined according the National Electrical Manufacturers Association (NEMA) standards 1-2008 with the expression SNR = S/N, where S = the signal and N = the noise of the image, which was estimated with a Rayleigh distribution (SD = standard deviation): N = SD/0.66. SNR values were measured from the b_0_ images in each region (b = 0 s/mm^2^).

Two experienced observers, a medical physicist (UH) and a neuroradiologist (AB), performed the freehand measurements on a workstation using commercially available software Neuro3D (Siemens Healthcare, Malvern, USA). The freehand ROIs were manually placed on the axial images of the color-coded FA maps and automatically transferred to the ADC, AD, and RD maps as well as the non-diffusion weighted b_0_ images. The ROIs were centered in the region using color-coded directions. The measurements were aimed to avoid border areas, such as areas overlapping with cerebrospinal fluid spaces, partial volume effects, and neighboring tracts. The thalamus was drawn to the grayscale FA map, because the border areas were more clearly distinguishable in this manner than in the color map.

**Fig. 1:**
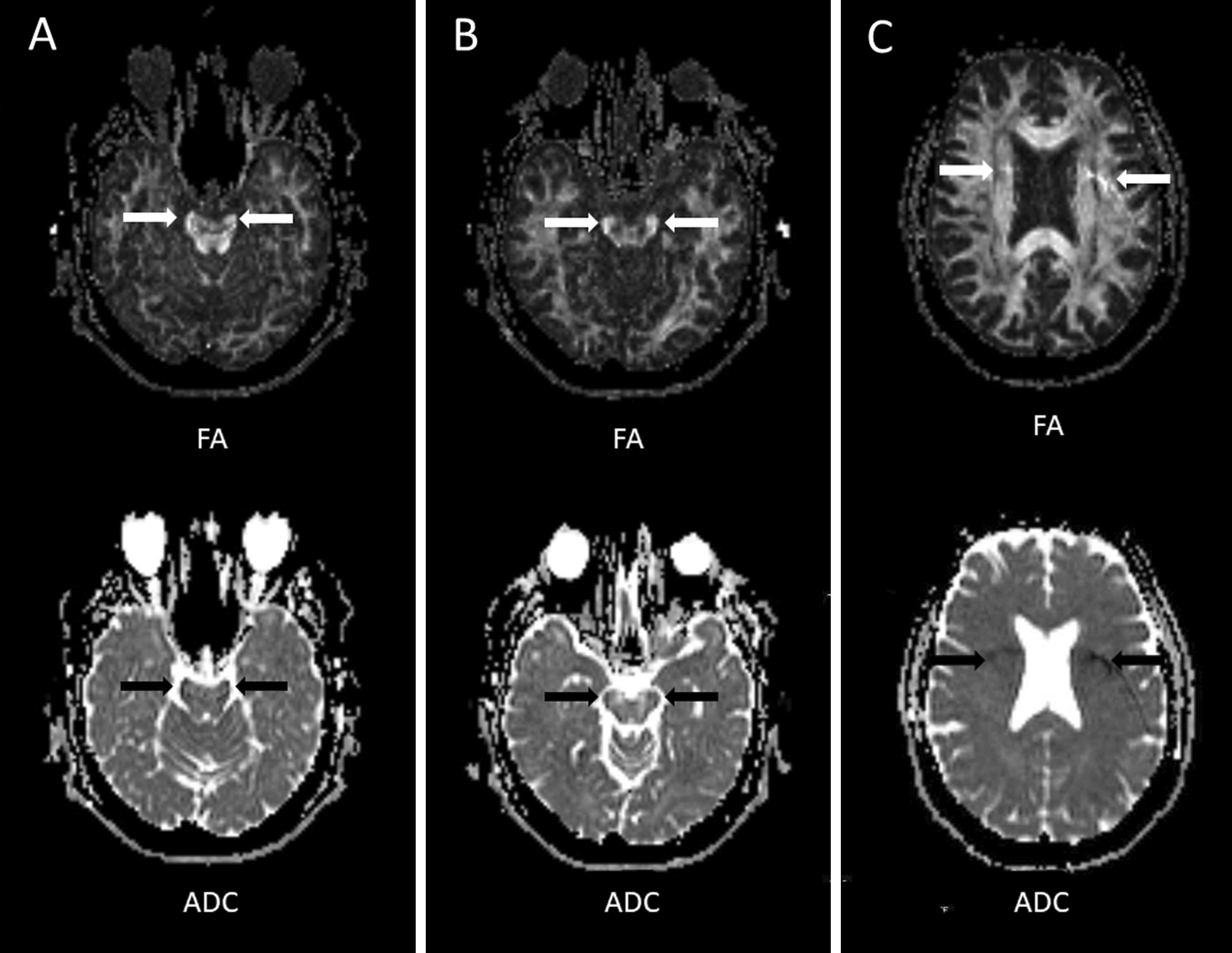
Axial FA and ADC maps with examples of common artifacts: **A** distortion in the cerebral peduncle, **B** susceptibility artifact (air-cavity) in the cerebral peduncle, and **C** phase artifact (fat misregistration) in the corona radiata

**Fig. 2:**
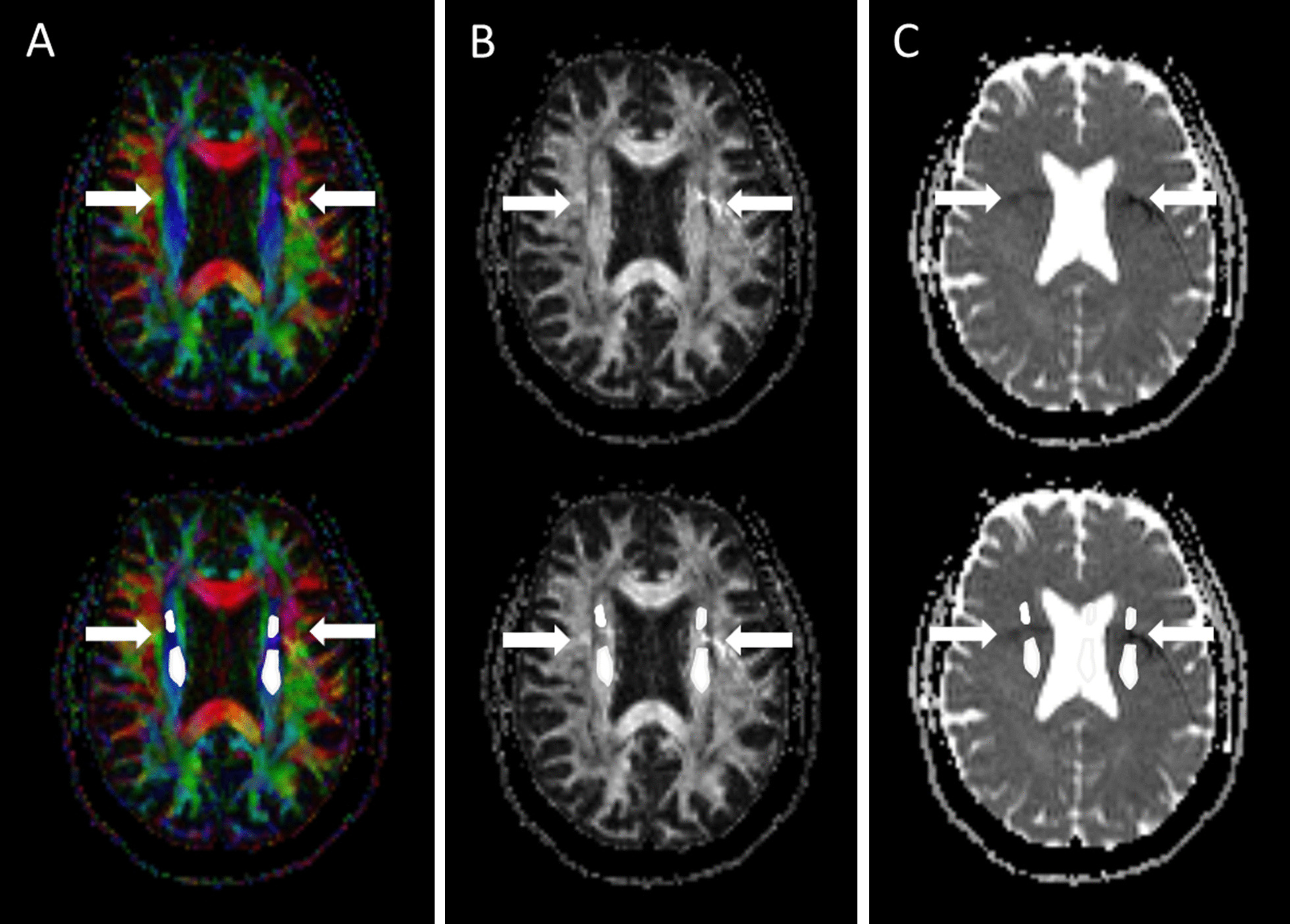
Examples of FA and ADC maps with a phase artifact (fat misregistration) in the corona radiata and how the artifact was excluded from the ROIs (ROIs marked in white): **A** axial FA color map, **B** axial FA map, and **C** axial ADC map

Slices containing artefacts were avoided. If this was not possible, the artefact areas were excluded by omitting them from the ROI regions (Figs. 1 and 2). The sizes of the ROIs were chosen using the anatomical knowledge of brain regions and a tract-based atlas of human white matter anatomy [43]. The ROI size ranged from 10 mm^2^ (min, cerebral peduncle) to 430 mm^2^ (max, centrum semiovale). The time between the first and repeated freehand ROI measurements was at least four weeks.

**Fig. 3:**
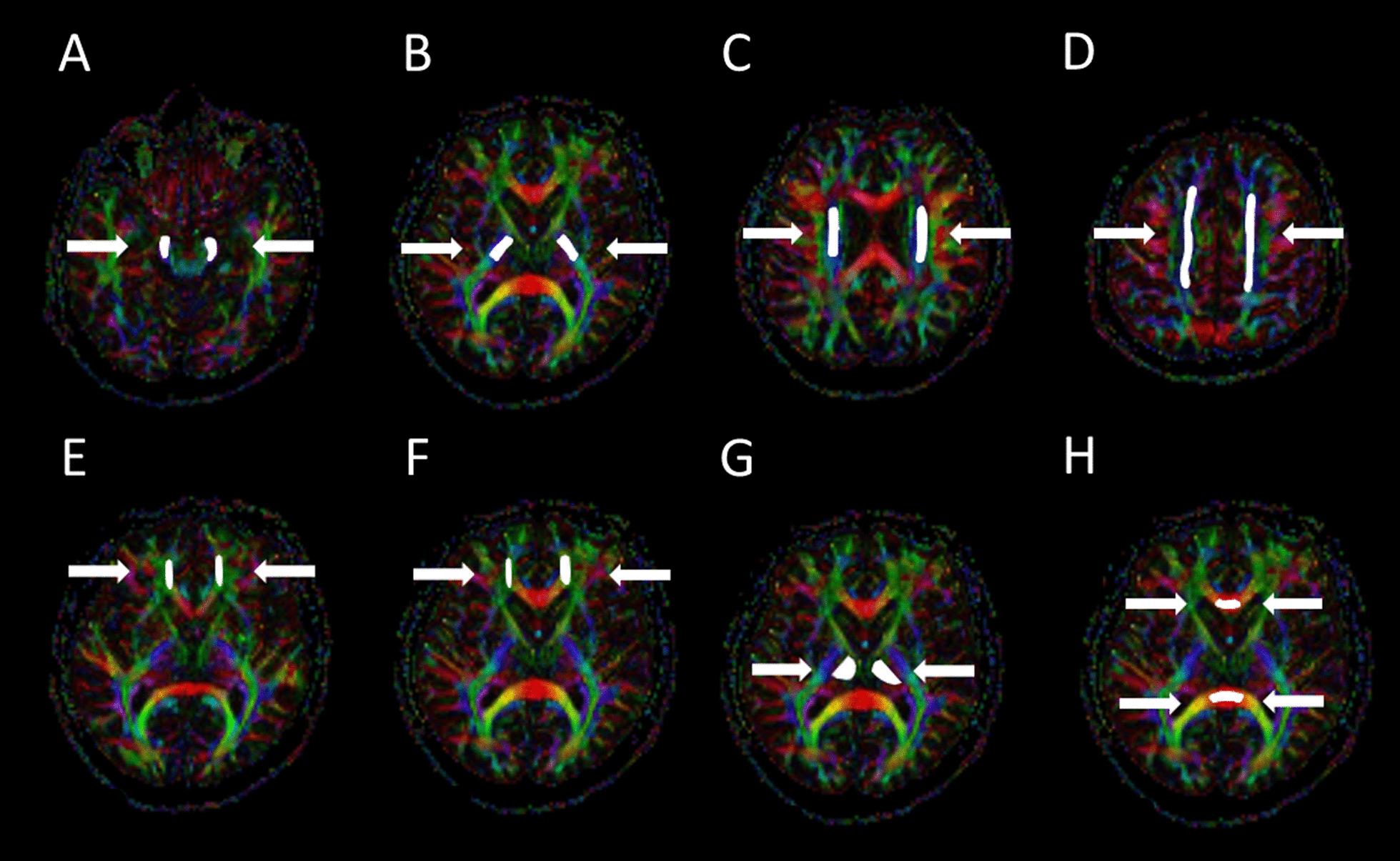
Axial FA color maps with the measured freehand ROIs (regions-of-interest) (ROIs marked in white): **A** cerebral peduncle, **B** posterior limb of the internal capsule, **C** corona radiata, **D** centrum semiovale, **E** uncinate fasciculus, **F** forceps minor, **G** thalamus, and **H** genu and splenium of the corpus callosum

Intra-observer measurements were performed for all volunteers (n = 40) and inter-observer measurements for 15 volunteers (n = 15). Nine regions were measured, eight of which were in the white matter (Fig. 3). Two observers analyzed each distinct region. The first observer (UH) analyzed the images of 40 subjects twice and the second observer (AB) measured images of 15 subjects. The same 15 subjects were selected from observer 1 measurements for inter-observer analysis. The measurements were selected from the first measurements. The regions in the pyramidal tracts included: the cerebral peduncle, posterior limb of the internal capsule, corona radiata, and centrum semiovale. In the frontobasal area, these included the uncinate fasciculus and forceps minor, while, in the corpus callosum, these included the genu and splenium. One region—the thalamus—was in the gray matter. The FA, ADC, AD, and RD values were calculated for each region. The left and right hemispheres were measured separately for seven regions. Moreover, the ROIs for the genu and splenium of the corpus callosum were drawn in the center of the axial image with one ROI per region.

### Statistical analyses

The statistical analyses were performed using the SPSS software package (IBM SPSS Statistics version 22 and 26, Chicago, IL). Means and standard deviations were calculated for each region and parameter, and asymmetries between hemispheres were evaluated using a paired samples t-test. The statistical significance was set to *p* < 0.007, with a Bonferroni correction for seven regions, according to the regions measured in each hemisphere of the brain. The normality of distributions was tested using the Shapiro–Wilk test (n < 50). The differences among all the age group means were analyzed using an analysis of variance (ANOVA) for the normally distributed data and Welch’s test in inhomogeneous cases, where the variance of the variable differed between the age groups. The Kruskal–Wallis test was used for non-normally distributed data. Correlation analysis between FA, ADC and age from the same data have been published in our previous study [41]. In that study, we mostly used a small circle ROI, including a freehand ROI in three regions for better repeatability.

The samples that showed statistically significant differences among the age groups were analyzed by a group comparison between the different age groups. The independent-samples t-test was used with the normally distributed samples, and the Mann–Whitney U test with the non-normal distributions.

To show the relative variability of each measurement, the percent coefficients of variation (CV%) were calculated according the following equation (with SD = standard deviation and M = mean): (SD/M) × 100% [44]. The variability was considered acceptable when the CV% was less than 10% [45]. The results between 11 and 20% were considered to be moderate but still adequate. CV% values over 21% were considered too high and inadequate.

Bland–Altman plots were used as graphical representations for intra- and inter-observer repeatability [44]. The 95% limits of agreement and ± 2 standard deviation of the differences were calculated. The better was consistency between the first and repeated measurements, the smaller the difference between the two limits. Intra- and inter-observer repeatability was also assessed using intra-class correlation coefficients (ICCs) with an absolute agreement. Two-way mixed option was chosen as the model because the aim was to investigate the repeatability of these specific observers. In this study, the average ICC refers to the repeatability (test–retest) when the same region is measured twice and the final score is the average of two measurements. The single ICC approximates a situation where the measurement would only be made once, as is usually the case in clinical situations. The cerebral hemispheres have been analyzed separately, but presented as the mean of the left and right hemispheres of the brain. The ICC values were considered to indicate excellent agreement if they were greater than 0.8. ICC results between 0.70 and 0.79 were considered adequate [45], and values below 0.69 were considered inadequate for clinical work. The statistical significance was set to *p* < 0.006, with a Bonferroni correction for nine regions.

## Results

The data quality was excellent in most cases. In some of the cases, artifacts were detected in the cerebral peduncle, corona radiata, and centrum semiovale (Table 1 and Fig. 2). Significant eddy current artefacts did not occur.

**Table 1:** The incidence of artifacts in the regions (N = 40)

Region		Geometric distortion (%)	Air-cavity (%)	Fat misregistration (%)
Cerebral peduncle	Right	17.5	65	
	Left	10.0	50	
Capsula Interna (posterior)	Right	–	–	7.5
	Left	–	–	7.5
Corona radiata	Right	–	–	0
	Left	–	–	55
Centrum semiovale	Right	–	–	5.0
	Left	–	–	25

The mean SNR values (± SD) for all regions was 27.7 ± 4.2: the pyramidal tract 30.5 ± 4.2, frontobasal area 24.1 ± 4.7, corpus callosum 25.4 ± 0.3, and thalamus 28.0 ± 4.2

### Mean values

In the Shapiro-Wilks test, 90% of the means were normally distributed (*p* > 0.05). The intra-observer mean values for the FA, ADC, AD, and RD of the sample (n = 40) are shown in Table 2.

**Table 2:** The intra-observer (observer 1) regional mean FA (0–1, unitless), ADC (10^−3^ mm^2^/s), AD (10^−3^ mm^2^/s) and RD (10^−3^ mm^2^/s) values ± standard deviation (mean ± SD), variation (the percent coefficients of variation = CV%) and repeatability (the intra-class correlation coefficients (ICC) and mean difference ± 2SD) (N = 40)

Region		1st meas	2nd meas	1st meas	2nd meas	Average	Single	1st and 2st meas
		Mean ± SD	Mean ± SD	CV (%)	CV (%)	ICC	ICC	Mean diff ± 2SD
Cerebral peduncle	FA	0.808 ± 0.041	0.804 ± 0.044	5.0	5.5	0.748	0.597	0.004 ± 0.077
	ADC	0.727 ± 0.045^a^	0.725 ± 0.051	6.2	7.0	0.801	0.668	0.002 ± 0.079
	AD	1.625 ± 0.106^b^	1.614 ± 0.122	6.5	7.6	0.859	0.752	0.010 ± 0.161
	RD	0.278 ± 0.051	0.280 ± 0.053	18.3	19.0	0.756	0.608	− 0.003 ± 0.093
Internal capsule (posterior)	FA	0.699 ± 0.033^c^	0.689 ± 0.037	4.7	5.3	0.859	0.752	0.010 ± 0.046
	ADC	0.706 ± 0.021^d^	0.702 ± 0.021	3.0	3.0	0.949	0.903	0.003 ± 0.018
	AD	1.388 ± 0.053	1.371 ± 0.055	3.8	4.0	0.877	0.781	0.018 ± 0.065
	RD	0.364 ± 0.030^e^	0.368 ± 0.034	8.2	9.2	0.912	0.839	− 0.004 ± 0.036
Corona radiata	FA	0.502 ± 0.049^f^	0.491 ± 0.047	9.7	9.6	0.867	0.766	0.011 ± 0.063
	ADC	0.700 ± 0.035^g^	0.708 ± 0.034	5.0	4.8	0.936	0.880	− 0.008 ± 0.030
	AD	1.104 ± 0.050	1.105 ± 0.048	4.5	4.3	0.848	0.736	− 0.001 ± 0.071
	RD	0.498 ± 0.048^h^	0.510 ± 0.050	9.7	9.8	0.917	0.846	− 0.012 ± 0.051
Centrum semiovale	FA	0.542 ± 0.067	0.549 ± 0.060	12.3	11	0.894	0.809	− 0.007 ± 0.078
	ADC	0.724 ± 0.030^i^	0.724 ± 0.029	4.2	4.0	0.976	0.953	0.000 ± 0.018
	AD	1.210 ± 0.066	1.217 ± 0.049	5.5	4.0	0.764	0.618	− 0.008 ± 0.102
	RD	0.481 ± 0.056	0.477 ± 0.052	11.7	11	0.938	0.882	0.004 ± 0.053
Uncinate fasciculus	FA	0.566 ± 0.060^j^	0.566 ± 0.056	10.6	9.9	0.945	0.895	− 0.001 ± 0.054
	ADC	0.784 ± 0.042	0.785 ± 0.041	5.3	5.3	0.954	0.912	− 0.001 ± 0.035
	AD	1.351 ± 0.078	1.355 ± 0.063	5.8	4.6	0.882	0.789	− 0.004 ± 0.093
	RD	0.500 ± 0.053	0.500 ± 0.059	10.5	11.8	0.963	0.929	0.000 ± 0.045
Forceps minor	FA	0.505 ± 0.058^k^	0.505 ± 0.054	11.5	10.6	0.933	0.874	0.001 ± 0.057
	ADC	0.771 ± 0.033	0.768 ± 0.034	4.3	4.5	0.940	0.887	0.003 ± 0.032
	AD	1.246 ± 0.071	1.240 ± 0.063	5.7	5.0	0.893	0.807	0.006 ± 0.084
	RD	0.533 ± 0.044	0.530 ± 0.053	8.3	10	0.946	0.897	0.003 ± 0.048
Thalamus	FA	0.316 ± 0.026	0.311 ± 0.028	8.3	9.2	0.862	0.758	0.005 ± 0.037
	ADC	0.758 ± 0.027	0.760 ± 0.027	3.5	3.5	0.960	0.924	− 0.001 ± 0.021
	AD	1.004 ± 0.033	1.001 ± 0.035	3.2	3.5	0.914	0.842	0.003 ± 0.038
	RD	0.635 ± 0.032	0.640 ± 0.029	5.0	4.5	0.917	0.847	− 0.004 ± 0.032
CC Genu	FA	0.861 ± 0.040	0.864 ± 0.045	4.6	5.3	0.855	0.747	− 0.002 ± 0.061
	ADC	0.757 ± 0.045	0.759 ± 0.047	6.0	6.2	0.829	0.708	− 0.001 ± 0.071
	AD	1.822 ± 0.100	1.831 ± 0.101	5.5	5.5	0.886	0.796	− 0.010 ± 0.129
	RD	0.225 ± 0.057	0.222 ± 0.064	25.4	28.7	0.857	0.750	0.003 ± 0.086
CC Splenium	FA	0.853 ± 0.043	0.847 ± 0.041	5.0	4.8	0.877	0.781	0.006 ± 0.055
	ADC	0.741 ± 0.057	0.748 ± 0.062	7.8	8.3	0.921	0.854	− 0.007 ± 0.064
	AD	1.768 ± 0.131	1.766 ± 0.138	7.4	7.8	0.942	0.891	0.001 ± 0.127
	RD	0.230 ± 0.060	0.238 ± 0.058	26.1	24.4	0.861	0.756	− 0.009 ± 0.082

FA, fractional anisotropy; ADC, apparent diffusion coefficient; AD, axial diffusivity; RD, radial diffusivity; ICC, intraclass correlation coefficient; CV, coefficient of variation; CC, corpus callosum; diff, difference; SD, standard deviation

Regions with significant differences between right (R) and left (L) cerebral hemispheres (*p* < 0.007):

^a,b^Cerebral peduncle (a) ADC: R 0.746 ± 0.062, L 0.707 ± 0.060 (b) AD: R 1.660 ± 0.138 L 1.592 ± 0.122;

^c,d,e^Posterior limb of the Internal capsule: (c) FA: R 0.693 ± 0.037, L 0.707 ± 0.036 d) ADC: R 0.713 ± 0.025, L 0.698 ± 0.024

(e) RD: R 0.373 ± 0.037, L 0.355 ± 0.031; (f,g,h) Corona radiata: (f) FA: R 0.693 ± 0.037, L 0.707 ± 0.036

(g) ADC: R 0.713 ± 0.025, L 0.698 ± 0.024 (h) RD: R 0.373 ± 0.037, L 0.355 ± 0.031

^i^Centrum Semiovale (i) ADC: R 0.731 ± 0.034, L 0.717 ± 0.032, ^j^Uncinate fasciculus (j) FA: R 0.575 ± 0.062, L 0.556 ± 0.064

^k^Forceps minor (k) FA: R 0.519 ± 0.070, L 0.491 ± 0.058

In white matter ROIs, the mean FA value was 0.67. The lowest value was found in the corona radiata (0.50), and highest in the genu of the corpus callosum (0.86). The mean ADC value was 0.74 × 10^−3^ mm^2^/s, with lowest value being found in the corona radiata (0.70 × 10^−3^ mm^2^/s) and the highest in the uncinate fasciculus (0.78 × 10^−3^ mm^2^/s). The mean AD value was 1.44 × 10^−3^ mm^2^/s, with the lowest value being found in the corona radiata (1.10 × 10^−3^ mm^2^/s), and highest in the genu of the corpus callosum (1.82 × 10^−3^ mm^2^/s). The mean RD value was 0.39 × 10^−3^ mm^2^/s, with the lowest value being found in the genu of the corpus callosum (0.26 × 10^−3^ mm^2^/s) and the highest in the forceps minor (0.53 × 10^−3^ mm^2^/s). In the gray matter—the thalamus—the corresponding mean values were 0.32 for the FA, 0.76 × 10^−3^ mm^2^/s for ADC, 1.00 × 10^−3^ mm^2^/s for AD, and 0.64 × 10^−3^ mm^2^/s for RD.

Statistically significant differences between the right and left hemispheres (paired t test, *p* < 0.007) are expressed in Table 2, and the absolute mean values can be found in the table footnotes. In the pyramidal tract, more precisely in the posterior limb of the internal capsule and corona radiata, the FA values were significantly higher and RD values lower in the left hemisphere. The ADC values were lower in the left hemisphere in all four regions of the pyramidal tract. In the cerebral peduncle, the AD value was also lower in the left hemisphere. In both frontobasal regions, the FA values were significantly higher in the right hemisphere.

**Fig. 4:**
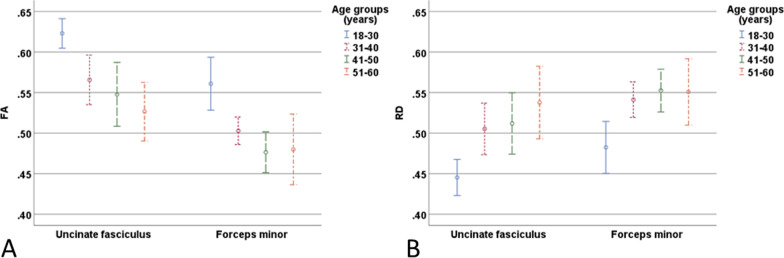
Sample images of significant differences between the youngest age group (18–30 years) and other age groups in the uncinate fasciculus and forceps minor: **A** FA (0–1, unitless), and **B** RD (10^−3^ mm^2^/s)

Significant differences between age groups were found in the frontobasal regions (Fig. 4). The FA and AD values were significantly higher and the RD values significantly lower in the youngest age group (18–30 years) compared to the other age groups (31–40, 41–50 and 51–60 years) (Fig. 4A, B). Specifically, the FA and RD differences were found in both hemispheres and AD differences in the left. For the ADC, there were no significant differences between the groups. The inter-observer mean values were estimated for 15 subjects, and the values are shown in Table 3.

**Table 3:** Inter-observer regional mean FA (0-1, unitless), ADC (10^−3^ mm^2^/s), AD (10^−3^ mm^2^/s) and RD (10^−3^ mm^2^/s) values ± standard deviation (mean ± SD) values, variation (the percent coefficients of variation = CV%) and repeatability (mean difference ± 2SD) (observer 1 & 2) (N = 15)

Region		Obs. 1	Obs. 2	Obs. 1	Obs. 2	Obs.1 and Obs. 2
		Mean ± SD	Mean ± SD	CV(%)	CV(%)	Mean diff ± 2SD
Cerebral peduncle	FA	0.817 ± 0.039	0.818 ± 0.033	4.8	4.1	− 0.001 ± 0.057
	ADC	0.731 ± 0.049	0.721 ± 0.051	6.7	7.1	0.010 ± 0.072
	AD	1.652 ± 0.115	1.632 ± 0.113	7.0	6.9	0.021 ± 0.139
	RD	0.271 ± 0.052	0.270 ± 0.048	19.2	17.7	0.000 ± 0.065
Internal capsule (posterior)	FA	0.707 ± 0.027	0.719 ± 0.040	3.8	5.6	− 0.012 ± 0.053
	ADC	0.709 ± 0.025	0.711 ± 0.028	3.5	4.0	− 0.002 ± 0.031
	AD	1.403 ± 0.043	1.435 ± 0.064	3.1	4.5	− 0.033 ± 0.101
	RD	0.360 ± 0.030	0.346 ± 0.040	8.3	11.5	0.014 ± 0.044
Corona radiata	FA	0.524 ± 0.049	0.522 ± 0.050	9.3	9.6	0.002 ± 0.049
	ADC	0.683 ± 0.038	0.686 ± 0.043	5.5	6.3	− 0.003 ± 0.028
	AD	1.099 ± 0.049	1.108 ± 0.055	4.4	5.0	− 0.008 ± 0.069
	RD	0.475 ± 0.052	0.477 ± 0.056	10.9	11.7	− 0.002 ± 0.036
Centrum semiovale	FA	0.571 ± 0.080	0.579 ± 0.054	14.1	9.3	− 0.008 ± 0.133
	ADC	0.716 ± 0.029	0.708 ± 0.035	4.1	4.9	0.009 ± 0.033
	AD	1.238 ± 0.082	1.299 ± 0.046	6.6	3.7	0.010 ± 0.182
	RD	0.456 ± 0.064	0.447 ± 0.051	14.1	11.3	0.009 ± 0.080
Uncinate fasciculus	FA	0.595 ± 0.057	0.562 ± 0.054	9.6	9.6	0.033 ± 0.033
	ADC	0.789 ± 0.050	0.786 ± 0.041	6.3	5.2	0.003 ± 0.043
	AD	1.403 ± 0.069	1.348 ± 0.065	4.9	4.9	0.054 ± 0.089
	RD	0.482 ± 0.065	0.505 ± 0.057	13.5	11.3	− 0.023 ± 0.035
Forceps minor	FA	0.529 ± 0.072	0.494 ± 0.069	13.5	14	0.034 ± 0.113
	ADC	0.768 ± 0.037	0.771 ± 0.044	4.8	5.7	− 0.002 ± 0.051
	AD	1.275 ± 0.073	1.234 ± 0.063	5.7	5.1	0.041 ± 0.126
	RD	0.515 ± 0.064	0.539 ± 0.066	12.5	12.3	− 0.024 ± 0.093
Thalamus	FA	0.322 ± 0.031	0.315 ± 0.027	9.5	8.5	0.006 ± 0.050
	ADC	0.755 ± 0.028	0.750 ± 0.028	3.7	3.8	0.005 ± 0.018
	AD	1.005 ± 0.031	0.992 ± 0.024	3.1	2.5	0.012 ± 0.054
	RD	0.630 ± 0.034	0.629 ± 0.033	5.4	5.3	0.001 ± 0.028
CC Genu	FA	0.868 ± 0.042	0.849 ± 0.057	4.8	6.7	0.019 ± 0.064
	ADC	0.745 ± 0.047	0.760 ± 0.055	6.3	7.2	− 0.015 ± 0.085
	AD	1.808 ± 0.093	1.799 ± 0.109	5.2	6.0	0.009 ± 0.186
	RD	0.213 ± 0.062	0.240 ± 0.081	29.1	33.7	− 0.026 ± 0.086
CC Splenium	FA	0.868 ± 0.050	0.862 ± 0.054	5.7	6.2	0.006 ± 0.092
	ADC	0.733 ± 0.067	0.749 ± 0.053	9.2	6.2	− 0.017 ± 0.098
	AD	1.778 ± 0.140	1.805 ± 0.112	7.9	6.2	− 0.027 ± 0.136
	RD	0.210 ± 0.074	0.222 ± 0.076	35.2	34.4	− 0.011 ± 0.136

FA, fractional anisotropy; ADC, apparent diffusion coefficient; AD, axial diffusivity; RD, radial diffusivity

CV, coefficient of variation; CC, corpus callosum; diff, difference; SD, standard deviation

### Variation

**Fig. 5:**
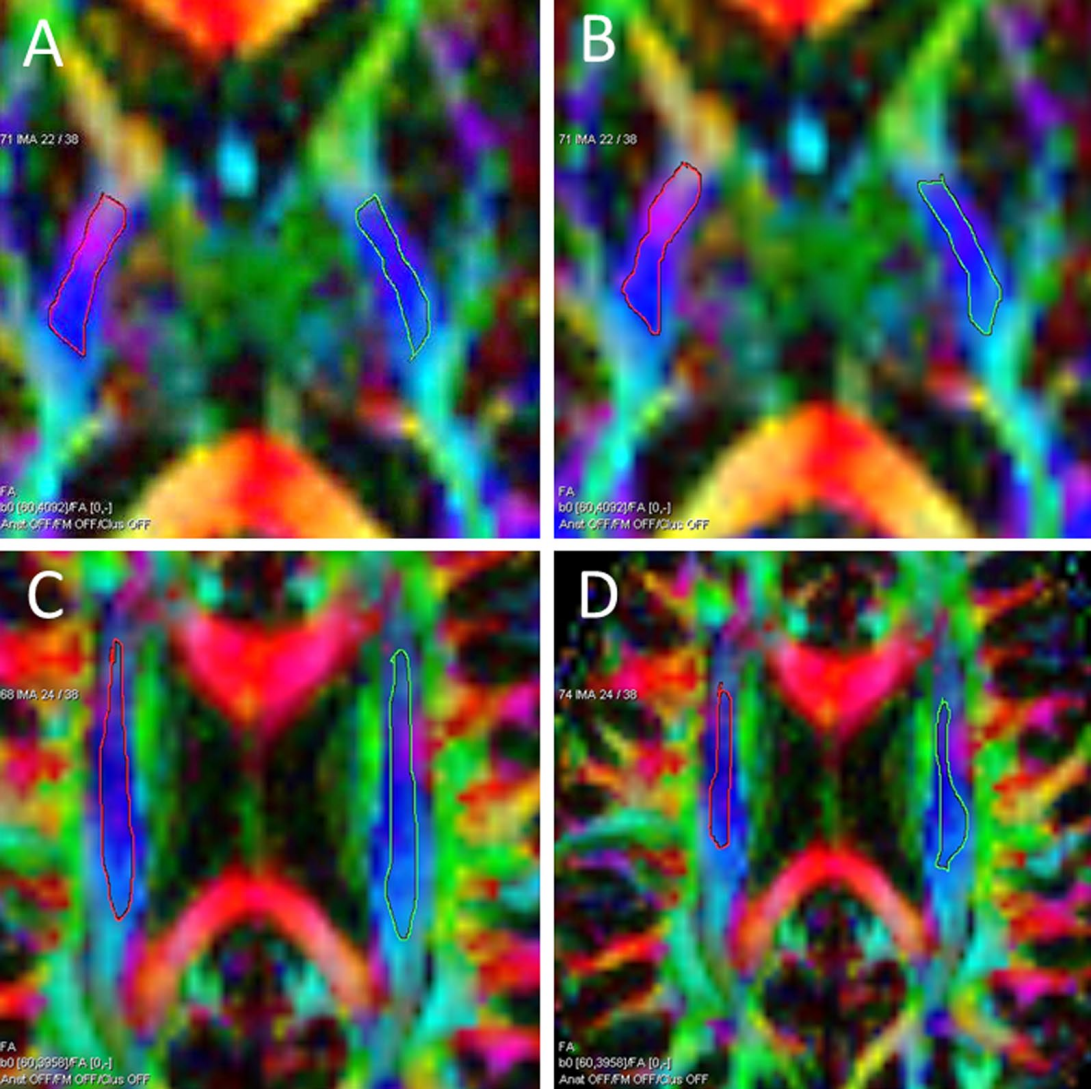
Examples of regions of interest variation between measurements. **A**, **B** The intra-observer measurement in the posterior limb of the capsula interna (observer 1): **A** the first measurement; **B** the repeat measurement. **C**, **D** The inter-observer measurement in the corona radiata: **C** observer 1; **D** observer 2

The intra-observer variations (CV%) are shown in Table 2 (n = 40) (Fig. 5A). In the pyramidal tract, the variation for the FA measurements was 8%. The lowest variation was in the posterior limb of the capsula interna (5%), and the highest in the centrum semiovale (12%). The variation was 11% in the frontobasal area and 5% in the corpus callosum. In the gray matter (thalamus), the variation for the FA was 8%. For the ADC and AD, it was between 3 to 8% with all white matter and gray matter regions. For the RD measurements, the variation in the pyramidal tract was 12%. The lowest variation was in the posterior limb of the capsula interna (8%) and the highest in the cerebral peduncle (18%). The RD variation was 9% in the frontobasal area and 26% in the corpus callosum. In the gray matter (thalamus), the variation was 5%. The inter-observer variation results (CV%) are shown in Table 3 (Fig. 5B).

### Reliability

**Fig. 6:**
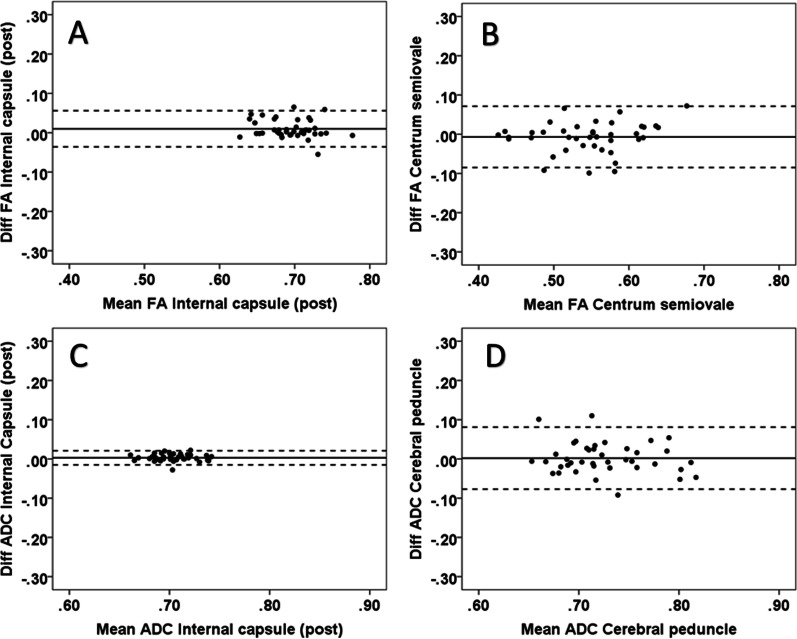
The intra-observer reliability for FA (scale 0–1, unitless) and ADC (10^-3^ mm^2^/s) in select white matter regions; the Bland–Altman plots show minimum and maximum differences with 95% limits of agreement (dotted lines). In the plots, the horizontal scales are the mean of two measurements and the vertical scales are ± 2SD (SD = standard deviation, diff = difference and post = posterior): **A** minimum difference in the posterior limb of the internal capsule (FA), **B** maximum difference in the cerebral peduncle (FA), **C** minimum difference in the posterior limb of the internal capsule (ADC), and **D** maximum difference in the cerebral peduncle (ADC)

**Fig. 7:**
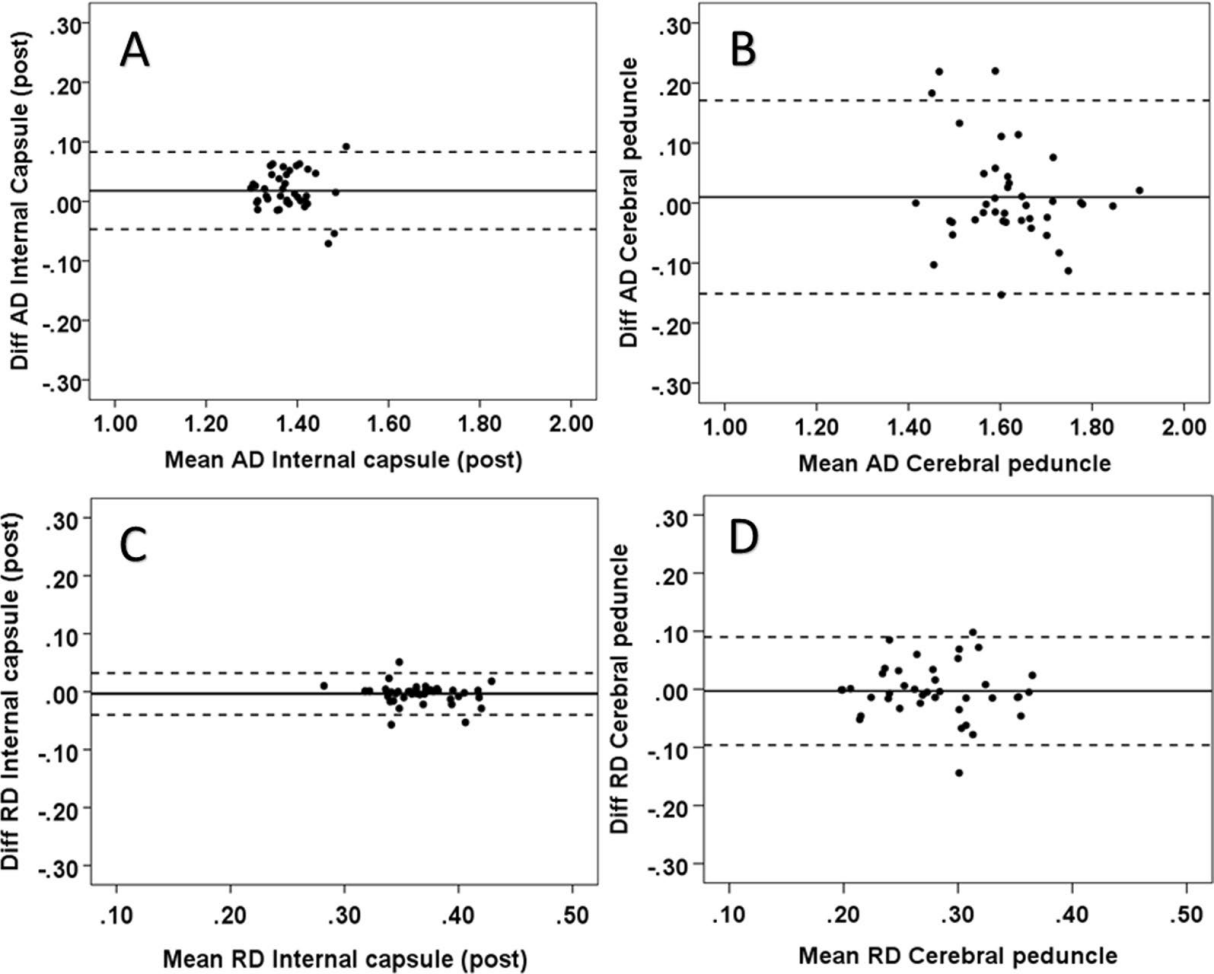
Intra-observer reliability for AD (10^−3^ mm^2^/s) and RD (10^−3^ mm^2^/s) in select white matter regions; the Bland–Altman plots show minimum and maximum differences with 95% limits of agreement (dotted lines). In the plots, the horizontal scales are the mean of two measurements and the vertical scales are ± 2SD (SD = standard deviation, diff = difference and post = posterior) **A** minimum difference in the posterior limb of the internal capsule (AD), **B** maximum difference in the cerebral peduncle (AD), **C** minimum difference in the posterior limb of the internal capsule (RD), and **D** maximum difference in the cerebral peduncle (RD)

**Fig. 8:**
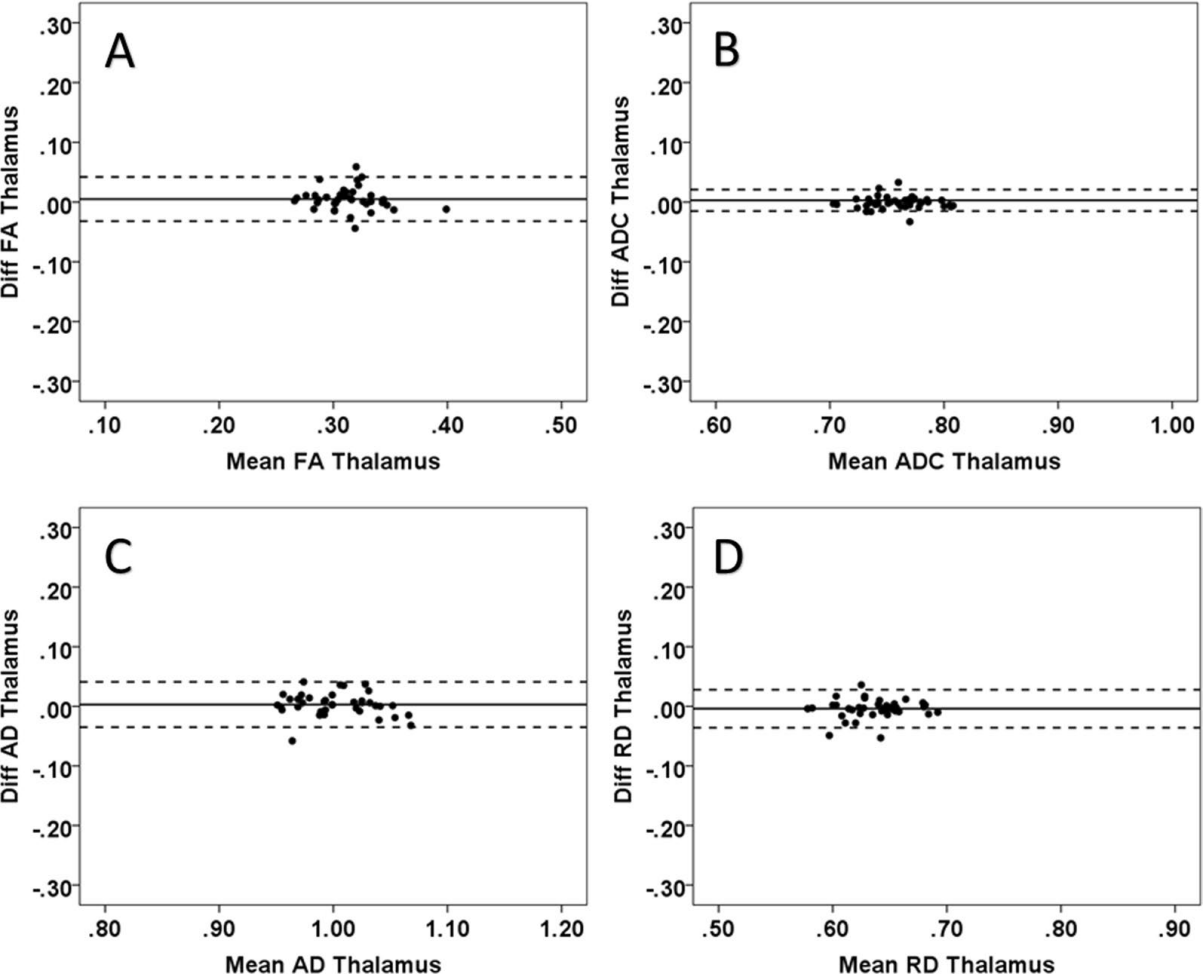
Intra-observer reliability for all parameters (FA (skale 0–1, unitless), ADC (10^−3^ mm^2^/s), AD (10^−3^ mm^2^/s) and RD (10^−3^ mm^2^/s) in the thalamus; the Bland–Altman plots show 95% limits of agreement (dotted lines). In the plots, the horizontal scales are the mean of two measurements and the vertical scales are ± 2SD (SD = standard deviation, diff = difference): **A** thalamus (FA), **B** thalamus (ADC), **C** thalamus (AD), and **D** thalamus (RD)

**Fig. 9:**
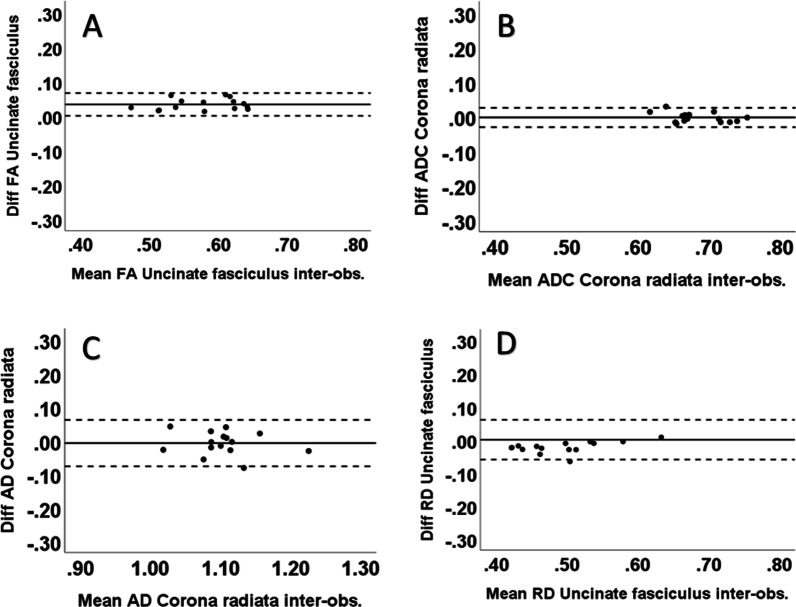
Inter-observer reliability; the Bland–Altman plots show minimum differences with 95% limits of agreement (dotted lines) with all parameters (FA (scale 0–1, unitless), ADC (10^−3^ mm^2^/s), AD (10^−3^ mm^2^/s) and RD (10^−3^ mm^2^/s). In the plots, the horizontal scales are the mean of two measurements and the vertical scales are ± 2SD (SD = standard deviation, diff = difference and post = posterior): **A** uncinate fasciculus (FA), **B** corona radiata (ADC), **C** corona radiata (AD), and **D** uncinate fasciculus (RD)

The intra-observer results of the limits of agreement are shown in Table 2. In the white matter, the best intra-observer agreement was found in the posterior limb of the capsula interna with all diffusion parameters. For the ADC, good agreement was also found in the corona radiata, centrum semiovale, uncinate fasciculus, and forceps minor. The largest range between the limits was found in the centrum semiovale for the FA and in the cerebral peduncle for the ADC, AD and RD measurements. The smallest and largest ranges between the 95% limits of agreement for each DTI parameter are presented in the Bland–Altman plots (Figs. 6, 7). For the gray matter, the agreement was very good with all DTI parameters (Fig. 8). On average, the 2 SD of the limit of agreement for the intra-observer results was 0.06. The inter-observer limits of agreement are shown in Table 3, and the smallest ranges between limits are presented in the Bland–Altman plots for each DTI parameter (Fig. 9). In white matter regions, the best agreement was found in the uncinate fasciculus for FA and RD in the corona radiata for ADC and AD. On average, the 2 SD of the limit of agreement for the inter-observer results was 0.08.

The intra-observer repeatability results (ICC) are shown in Table 2. For the FA, the mean was 0.87 for the average ICC and 0.78 for the single ICC. The highest average ICC was found in the uncinate fasciculus (0.95), and lowest in the cerebral peduncle (0.75). The average ICC results for the FA were above 0.8, and the single ICCs were above 0.7 in eight of the nine regions. Only one region, cerebral peduncle, had coefficients below these results (average 0.75 and single 0.60). For the ADC, the mean value for the average ICC was 0.91 and 0.85 for the single ICC. The highest ICC values were found in the centrum semiovale at both the average (0.98) and single (0.95) ICC. The lowest ICC was observed in the cerebral peduncle for both the average (0.80) and single (0.67) ICC. For AD, the mean average ICC result was 0.87, and the single ICC result was 0.78. The highest ICC values of AD were found in the splenium of the corpus callosum for both the average (0.94) and single (0.89). The lowest result of AD was in the centrum semiovale at the average (0.76) and single (0.62). For RD, the ICCs results were 0.90 for the average and 0.82 for the single measurement. The best repeatability values of ICCs for the average (0.96) and single (0.93) measurements were both found in the frontobasal area in the uncinate fasciculus. For RD, the lowest value was found in the cerebral peduncle by both the average result (0.76) and the single measurement (0.61). 70% of the inter-observer ICC results were statistically significant (*p* < 0.006). Only significant results were presented. The means of the average ICCs were 0.84 for FA, 0.88 for ADC, 0.81 for AD, and 0.88 for RD and the means of the single ICCs were 0.72, 0.79, 0.69 and 0.78, respectively. The highest ICCs were found in the corona radiata, the average ICC values were 0.94 for FA, 0.95 for ADC and 0.97 for RD and for the single ICCs 0.89, 0.90, 0.94, respectively. For AD, the highest ICCs were found in the splenium of the corpus callosum (the average 0.92 and single 0.84).

## Discussion

FA values are considered to reflect the integrity of the white matter. Although not in itself a specific parameter in a diagnostic sense, it provides indirect information about myelination, fiber packing density, and fiber orientation [46]. It is well-known that FA values vary widely at different anatomic levels of the brain [12, 13, 40, 45, 47]. Specifically, Lee et al. [12] reported that regional FA values varied from 0.21 in deep gray matter (putamen) to 0.81 in tightly packed parallel white matter tract bundles, such as the genu of the corpus callosum. The corresponding results in this study were 0.32 for deep gray matter (thalamus) and 0.86 for the genu of the corpus callosum. Regions with coherently oriented fibers, such as the cerebral peduncle, internal capsule, and corpus callosum exhibited higher anisotropy than regions with less coherence, such as the centrum semiovale and other subcortical regions [48]. Due to the vast regional variability of FA, possible anatomical mismatches should be taken into account in inter-observer and intergroup comparisons [47]. The ADC values, on the other hand, exhibit less regional variation [13]. In our study, the ADC mean values varied between 0.7–0.8 × 10^−3^ mm^2^/s, and in other similar studies the variation was 0.7 to 0.9 × 10^−3^ mm^2^/s [45, 49–51]. In the frontobasal area, compared to other white matter regions demonstrated lower FA and AD values and higher ADC and RD values. The FA values were in line with a tractography study by Deng et al. [52], where a mean FA value of 0.41 (profile 0.3 to 0.52) was found in the uncinate fasciculus and 0.54 (profile 0.40 to 0.68) in the forceps minor. In our study, the corresponding FA values were 0.57 and 0.51. The results of Lieberman et al. [53] were also similar to ours in the uncinate fasciculus. The FA and ADC values were almost identical to those found in our previous study (30 subjects) in most of the regions [40]. The biggest difference (14%) between our present and previous study was found in the genu of the corpus callosum. In this region, measurements were previously made on sagittal [40] instead of axial images, like in the present study. In general, the measured quantitative diffusion metrics were well in line with previous studies.

Asymmetry between the hemispheres was found in some of the regions. Pyramidal tracts, such as the posterior limb of the capsula interna and corona radiata, expressed higher FA values and lower ADC and RD values in the left hemisphere. The present results are well in agreement with previous studies [13, 40, 54]. In addition, in the centrum semiovale, asymmetry of the cerebral hemispheres was observed in the ADC value, which was also lower on the left. Some of the observed asymmetry in our study may be attributed to handedness of the volunteers; 39 of the 40 volunteers in our study were right-handed. Corresponding hemispheric differences were obtained for right-handers in another study [54]. Phase artifacts (fat misregistration) could also be a possible explanation in the regions of the corona radiata and centrum semiovale. In the corona radiata, phase artifacts were present in 55% of cases in the left hemisphere but were not present at all in the right hemisphere. Similarly, the centrum semiovale included artifacts in 25% of cases in the left hemisphere and only in 5% in the right hemisphere. The fat misregistration generally raises FA values locally and decreases ADC and RD values. Artifacts can affect the ROIs in the vicinity, even if the visible part of the artifact is cropped out. Hemispheric differences were also found in the frontobasal area. In those regions, the FA values were found to be higher in the right hemisphere, which is in agreement with previous findings [40, 55]. Jahanshad et al. [55] found that the variance in the asymmetry of the frontal lobe is strongly due to genetic factors. In our study, higher FA values were usually found in the right hemisphere of the frontobasal area. Bonekamp et al. [56] reported that small hemispheric differences could be due to slight slice angulation. Therefore, keeping the same slice position and orientation in longitudinal studies is essential [47].

In terms of age-related changes, we found significant differences between the youngest age group (18–30 years) and other age groups (31–40, 41–50, and 51–60 years). Specifically, the FA values were higher and the RD values lower in the frontobasal area in both hemispheres in the youngest age group when compared to the other age groups. For FA, this result has already been published in our previous study [41]. Other studies have also found changes in the frontal regions of the brain caused by aging [16, 17]. In general, several studies have found a negative correlation between age and FA and a positive correlation between age and RD in white matter [21, 22, 57, 59]. These variations may be related to changes in myelination and axon density [17, 58, 60].

In the present study, acceptable intra-observer variability (≤ 10%) was found in six out of nine regions for FA, while three regions had moderate but adequate variation. For ADC and AD, all regions had acceptable variability. For RD, seven out of nine regions had an acceptable or moderate variation and two had high variation (genu and splenium of the corpus callosum). The percent variation of the RD values in the corpus callosum is naturally high, because the mean value is clearly lower than in the other regions. Low RD values are due to the fact that the fibers are tightly packed and parallel to each other. In this case, the variation was not a good indicator for assessing reliability. Overall, the variation results were in line with our previous study [40]. It is noteworthy that the freehand method gives an average of 4% lower variations in the pyramidal regions compared to the circle method [13, 41]. In contrast, in our study, the freehand method gave a slightly higher variation in the corpus callosum than the circle method in previous studies [13, 41]. This may be due to the fact that in our study, ROIs were plotted on the axial image, whereas in previous studies they were plotted on the sagittal image [13, 41]. Thus, in this particular region, it would be better to use the circle method for a sagittal image than the freehand method for an axial image. The inter-observer (n = 15) variability was acceptable or moderate in seven out of nine regions. The inter-observer variabilities are in line with our previous study [40].

The intra-observer repeatability was at a very good level according to the 95% limits of agreement. The results varied according to region, and, with tightly packed white matter tracts, such as the posterior limb of the capsula interna, the difference between the limits was small. Also, the only region of gray matter—the thalamus—was found to be reliable in this analysis. Furthermore, this difference was greater in regions containing crossing fibers, such as the centrum semiovale. Overall, the results were consistent with our previous research [40]. The inter-observer agreement was lower than the intra-observer agreement in all regions, and others have reported similar results [13, 40, 59, 60]. Several studies have shown that inter-observer agreement results have been one-third lower than intra-observer results [59, 60]. Our study further confirms the trend between inter-observer and intra-observer agreements. The uncinate fasciculus was found to be the most reliable region in the inter-observer analyses for FA and RD, while the corona radiata was the most reliable region for ADC and AD.

The intra-observer reliability was high according to the average measures of the ICC analysis. In our study, average ICC refers to the repeatability obtained as the average of two measurements from a single region. Overall, the average ICC results were excellent for all four parameters. The repeatability result was also excellent (above 0.8) in eight out of nine regions for FA and all regions for the ADC. The repeatability of the freehand method was significantly improved compared to our previous study [40]. The average ICC increase was 0.4 (37%) in terms of the FA and ADC parameters.

The higher ICC values were probably due to increased observer experience in selecting a slide, avoiding artifacts and the partial volume effect of border areas. The single intra-observer ICC analysis was, on average, excellent in terms of the ADC and RD parameters and moderate in terms of the FA and AD parameters. Single ICC in our study refers to the repeatability of a single measurement, which can be considered normal practice in clinical measurements. The results showed excellent or moderate repeatability in seven out of nine regions for all DTI parameters. The region with the highest single ICC values was the forceps minor, with excellent reliability for each parameter. Good reliability was also found in the following regions: the uncinate fasciculus, thalamus, and the genu and splenium of the corpus callosum. High reliability in the corpus callosum is consistent with previous studies with the ROI method [45, 61, 62] but also with the TBSS method [38]. Inadequate results (ICC < 0.69) were found in the cerebral peduncle (FA, ADC and RD) and centrum semiovale (AD). The reason for the inferior reliability of the cerebral peduncle was the susceptibility artifact, more specifically the air-cavity. This artifact causes local changes in the results of the parameters. Although efforts were made to avoid distracted areas in the ROI, the effects of the artifact were also reflected in the surrounding areas. The reason for the low reliability of the centrum semiovale in the AD values can be explained by the multitude of crossing fibers in the subcortical white matter. Also, the statistically significant inter-observer results were highly similar to the intra-observer results. The differences between intra- and inter-observer ICC results averaged at less than 5% for the average ICC and less than 10% for the single ICC. The most reliable inter-observer region was found to be the corona radiata, which had the highest value for three different parameters (FA, ADC, and RD). For AD, the highest value was obtained in the splenium of the corpus callosum. The reliability of the measurements is greatly improved if the measurement is repeated at least once or if the result is taken as a mean of the measurements from two different observers.

The SNR measurements showed that the image quality was sufficient for reliable quantitative measurements. In general, the SNR of b = 0 s/mm^2^ should be at least 20 in order to derive reliable FA values [36]. In our study, the SNR was well above 20 in all regions, and the measured SNR values were comparable to other studies [63, 64].

A limitation of this study was that the commercial program did not include eddy current and subject motion corrections. In addition, the used imaging parameters may have not been optimal, especially compared to more recent diffusion imaging, e.g., high angular resolution diffusion imaging (HARDI) using isotropic voxels. Acquisition with higher resolution isotropic voxels and possibly HARDI may give more accurate results [36]. Furthermore, it has been shown that using near 1 mm isotropic voxels gives excellent results in repeatability [65]. In addition, 70% of the inter-observer ICC results were statistically significant. This was a consequence of the small number of samples. The schedule of measurements was limited.

In general, the regions with high reliability and low variation possess some common features. These regions have low anatomical variation and tightly packed fibers with a common orientation [66]. These areas also often have a better SNR, fewer partial volume effects, and are also less affected by “crossing” fibers. In addition, the larger ROI size increases the SNR value and improves the repeatability [66]. When a larger ROI size is used in a limited region, it is likely that there are more percentages of the same voxels between the two measurements than for a smaller ROI. The results of the repeat measurements are thus close to each other.

In future studies, larger samples of carefully collected high-spatial and -angular resolution DTI normal data should be acquired. In those studies, more subjects should be recruited for each age group in order to perform a reliable analysis of the effect of age. In addition, it would be interesting to study how much the reliability of the measurements improve when different methods, such as the ROI, tractography, and TBSS, are used simultaneously.

## Conclusions

According to our results, the intra-observer repeatability of the quantitative freehand ROI method can be considered at least adequate. The quantitative freehand ROI method can be considered highly reliable for the average ICC and mostly adequate for the single ICC. The reliability of the single measurements was excellent or moderate in 80% of the regions, including all DTI parameters. In the comparison of parameters, for the single ICCs, most of the repeatability results were excellent in terms of the ADC and RD while only moderate in terms of the FA and AD parameters.

As per our results, the freehand method can be considered highly suitable for research and clinical applications assuming a well-experienced observer. Measurements should be repeated at least once in each region to ensure sufficient reliability of the results. The frontobasal area, such as the uncinate fasciculus and forceps minor, as well as the internal capsule and corona radiata regions of the pyramidal tracts were found to be reliable regions in the repeatability analysis. In addition, the only region of gray matter—the thalamus—was found to be reliable. Therefore, they could be considered as regions which yield the most accurate quantitative ROI measurements in clinical settings. In general, it would be highly beneficial to favor regions with high reliability and repeatability in ROI measurements, if possible. Additionally, special care should be taken in ROI delineation in subjects with image artifacts.

When using the results of healthy adults as a control for patient groups, it should be noted that the results are most reliable on adults less than 30 years of age whose brain white matter does not yet have age-related changes.

## Data Availability

The datasets used and/or analysed during the current study are available from the corresponding author on reasonable request.
